# An aptamer-based depot system for sustained release of small molecule therapeutics

**DOI:** 10.1038/s41467-023-37002-0

**Published:** 2023-04-28

**Authors:** Dali Wang, Yang Li, Xiaoran Deng, Matthew Torre, Zipei Zhang, Xiyu Li, Wei Zhang, Kathleen Cullion, Daniel S. Kohane, Christopher B. Weldon

**Affiliations:** 1 Laboratory for Biomaterials and Drug Delivery, Department of Anesthesiology, Boston Children’s Hospital, Harvard Medical School, Boston, MA 02115 USA; 2 Department of Surgery, Boston Children’s Hospital, Harvard Medical School, Boston, MA 02115 USA; 3 Department of Pathology, Brigham and Women’s Hospital, Harvard Medical School, Boston, MA 02115 USA; 4 Department of Pediatric Oncology, Dana-Farber Cancer Institute, Harvard Medical School, Boston, MA 02115 USA

**Keywords:** DNA nanotechnology, Drug delivery, Pain management, Nanomedicine, Therapeutics

## Abstract

Delivery of hydrophilic small molecule therapeutics by traditional drug delivery systems is challenging. Herein, we have used the specific interaction between DNA aptamers and drugs to create simple and effective drug depot systems. The specific binding of a phosphorothioate-modified aptamer to drugs formed non-covalent aptamer/drug complexes, which created a sustained release system. We demonstrated the effectiveness of this system with small hydrophilic molecules, the site 1 sodium channel blockers tetrodotoxin and saxitoxin. The aptamer-based delivery system greatly prolonged the duration of local anesthesia and reduced systemic toxicity. The beneficial effects of the aptamers were restricted to the compounds they were specific to. These studies establish aptamers as a class of highly specific, modifiable drug delivery systems, and demonstrate potential usefulness in the management of postoperative pain.

## Abstract (web summary)

Controlled delivery of small molecule therapeutics is challenging. Here the authors report a simple and effective aptamer-based depot system where the formation of aptamer/drug complexes leads to sustained release, and using this approach demonstrate improved in vivo delivery of channel blockers.

## Introduction

Aptamers are single-stranded oligonucleotides that form tertiary conformations with short double-stranded regions through intramolecular base pairing that can bind non-covalently to small molecules with high affinity and specificity^1–3^. Their affinity and selectivity to targets rivals that of antibodies, and they do not have demonstrable biological toxicity^1,4^. They also have been shown to have limited or no immunogenicity^5^. Furthermore, since they are derived from random synthetic libraries of oligonucleotides, they do not have sequence homology to coding or non-coding genetic sequences of any organism^2^. These attributes have enabled aptamers to be utilized in a wide range of applications, including diagnostics^6,7^, biosensor technologies^8^, affinity isolation^9^, biomarker discovery^10^ and targeted therapeutics^11–13^. Aptamers have been used as the targeting ligands for a variety of systemically delivered drug delivery systems^14–17^. An aptamer-based systemic drug delivery system has also been described where the aptamer targeting specific tissues was loaded with drug due to the latter’s non-specific interaction with DNA^18^. This approach is presumably limited to drugs that can interact with aptamers by nonspecific interactions such as charge, hydrophobicity, etc. A much broader range of compounds could be used if the specific binding were the basis of interaction between drug and aptamer.

We have hypothesized that the aptamers’ ability to specifically bind molecules would allow them to act as drug delivery systems (DDS). Using the aptamer specificity for drug binding would likely obviate the ability to specifically target tissues. However, in depot-type applications the noncovalent binding of drugs would prolong duration of release (and therefore duration of effect), while reducing systemic toxicity.

Here, we test the hypothesis that aptamers can act as a depot DDS in an application where extension of effect is desirable and systemic toxicity from rapid release can be problematic: local anesthesia with the site 1 sodium channel blockers (S1SCBs) tetrodotoxin (TTX) and saxitoxin (STX). S1SCBs are ultra-potent local anesthetics that act by blocking the voltage-gated Na channel, but their durations of effect are relatively short^19^ and their clinical use is limited by significant systemic toxicity^20^. Their duration of effect can be improved by drug delivery techniques that prevent systemic release^21–23^, as can their systemic toxicity, which can otherwise be considerable. The hydrophilicity of S1SCBs makes physical encapsulation challenging. We postulated that the resulting aptamer/S1SCBs non-covalent complexes could be used as a sustained drug release system for TTX and STX.

Non-covalent complexation with aptamers greatly prolonged the duration of local anesthesia from S1SCBs and reduced their systemic toxicity. These benefits accrued to the compounds to which the aptamers were specific, and not to compounds that had similar actions but different structures. In principle, using this approach aptamers could be used to create drug delivery systems for a wide range of drugs.

## Results

### Design of chemically modified aptamer for TTX binding and control release

**Fig. 1:**
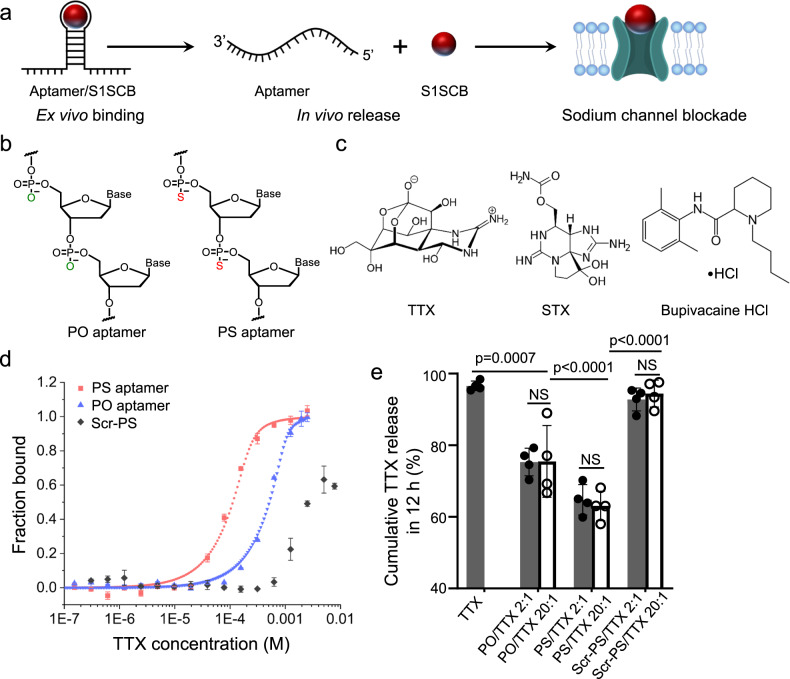
Aptamer for drug delivery. **a** Schematic of aptamer binding of drug, (site 1 sodium channel blocker [S1SCB]), then release in vivo where drug has effect. **b** Chemical structures of subunits of S1SCB-binding aptamers. PO: phosphodiester; PS: phosphorothioate. **c** Chemical structures of tetrodotoxin (TTX), saxitoxin (STX) and bupivacaine hydrochloride (HCl). **d** TTX binding affinity analysis by microscale thermophoresis of TTX-binding aptamers (PO and PS), and an aptamer with a scrambled sequence (Scr-PS). *Y*-axis shows the fraction of TTX bound to aptamer. *n* = 3 independent experiments. **e** Cumulative TTX release from aptamer/TTX complexes and controls at 12 h. The TTX concentration for each group was 42 μM. Data are the mean ± s.d., *n* = 4 independent experiments. Statistical analysis was performed using one-way ANOVA with Tukey’s multiple comparison testing. NS, not statistically significant. Source data are provided as a Source Data file.

To examine the feasibility of this concept, a known high-affinity TTX-binding aptamer (5ʹ-AAAAATTTCACACGGGTGCCTCGGCTGTCC-3ʹ) was chosen to create a non-covalent complex with TTX^24^ (Fig. 1a and Supplementary Table 1). To enhance resistance of the aptamer to nucleases, the phosphodiester (PO) backbone of aptamers was chemically modified with phosphorothioate (PS)^25,26^ (Fig. 1b and Supplementary Table 1).

To assess the binding affinity of TTX (Fig. 1c) to the PS aptamer^27^, interactions between TTX (in PBS) and a Cy5-labelled aptamer (Supplementary Table 1) were studied by microscale thermophoresis (MST), allowing determination of K_d_ values. PS modification improved the binding affinity of aptamer to TTX by a factor of 2, from a K_d_ of 29.5 ± 2.1 μM for the unmodified PO aptamer to 14.3 ± 1.7 μM (Fig. 1d). A PS aptamer containing a scrambled sequence (Scr-PS) (Supplementary Table 1) exhibited very limited measurable binding with TTX (K_d_ = 3.84 mM; ~270-fold greater [worse] than with PS), demonstrating that the specific aptamer sequence was mainly responsible for the observed binding.

To test if this binding affinity could be the basis of a sustained release system, TTX was complexed with aptamers (PO or PS) in a 2:1 or 20:1 molar ratio (aptamer:TTX) by simple mixing, and release kinetics of TTX from these formulations were investigated. These samples exhibited very similar mean diameters ~3 nm measured by dynamic light scattering (DLS) (Supplementary Fig. 1), indicating that association with TTX did not cause aggregation of aptamers. The release kinetics of TTX were studied by dialyzing 200 μL of aptamer/TTX (the concentration of TTX was 42 μM; the aptamer concentration varied) against 14 mL of PBS. The release of TTX was quantified by enzyme-linked immunosorbent assay (ELISA). Both PO/TTX and PS/TTX complexes (2:1 or 20:1) increased the duration of TTX release compared to free TTX (*p* < 0.0001 at 24 h, Fig. 1e and Supplementary Fig. 2). Release from PS/TTX was statistically significantly slower than from PO/TTX at both 12 h (*p* < 0.0001) and 24 h (*p* = 0.026). There was no statistically significant difference in TTX release between the two molar ratios tested (2:1, 20:1), for PO/TTX or PS/TTX (*p* > 0.05). To verify that the sequence is essential for aptamer interactions with TTX, the release kinetics of the non-selective scrambled PS aptamer (Scr-PS; Supplementary Table 1) affixed to TTX (Scr-PS/TTX) in two different molar ratios 2:1 and 20:1 were evaluated. Release of TTX from Scr-PS/TTX was more rapid than from PS/TTX, with 92.8 ± 3.6% (2:1) and 94.4 ± 3.8% (20:1) of TTX released in 12 h, which were similar to the rate of release of TTX without aptamer (*p* > 0.05 for all comparisons). These data demonstrate that the interaction between aptamer and TTX was sequence specific, and that the PS modification slowed release compared to PO. We attribute the slower release from PS/TTX to the improved binding affinity between the PS aptamer and TTX.

The cytotoxicity of aptamer/TTX was tested in two cell lines relevant to local anesthetic-related tissue injury: the myoblast C2C12 cell line was used to assess potential myotoxicity and the pheochromocytoma PC12 cell line was used to assess potential neurotoxicity^28,29^. Cells were incubated with free TTX, PO aptamer, PS aptamer, PO/TTX (2:1) complex, or PS/TTX (2:1) complex at the same TTX concentration of 73 μM and/or the aptamer concentration of 146 μM for 24 h and the cell viability was measured with the 3-(4,5-dimethylthiazol-2-yl)-5-(3-carboxymethoxyphenyl)-2-(4-sulfophenyl)-2H-tetrazolium) (MTS) assay^20^. All groups in both cell types maintained cell viability >90% compared with untreated cells (Supplementary Fig. 3).

### Comparing the effectiveness of free TTX and aptamer/TTX complexes on rat sciatic nerve blockade in vivo

To explore the feasibility of aptamers as a drug delivery system in vivo, we investigated the ability of aptamer to extend the duration of rat sciatic nerve block^23,30,31^. Male Sprague-Dawley rats (*n* = 4 or 6 per group) were injected at the left sciatic nerve with 0.3 mL of TTX alone or combined with an aptamer (PO, PS, or Scr-PS)/TTX. As 4 µg (42 µM) of free TTX in PBS provides nerve block without lethality in rats^30^, we set that as the initial TTX dose for all formulations. We first tested the aptamer (PO or PS)/TTX complexes at a fixed aptamer: TTX molar ratio of 20:1^30^. After injection, the rats underwent neurobehavioral testing to determine the duration of functional deficits (sensory and motor nerve blockade) in both hind paws^30,32^. Thermal latency (the time in seconds that the rat leaves its hindpaw on the hotplate) was the primary metric for sensory nerve block. A sensory nerve block was considered successful if the latency was longer than 7 s. The duration of the sensory nerve block was defined as the time for thermal latency to return to 7 s (the midpoint between baseline (2 s) and maximal latency (12 s)). Deficits in the left (ipsilateral) hindpaw reflect nerve block, while deficits in the right (contralateral) hindpaw reflect systemically distributed drug (i.e., systemic toxicity).

**Fig. 2:**
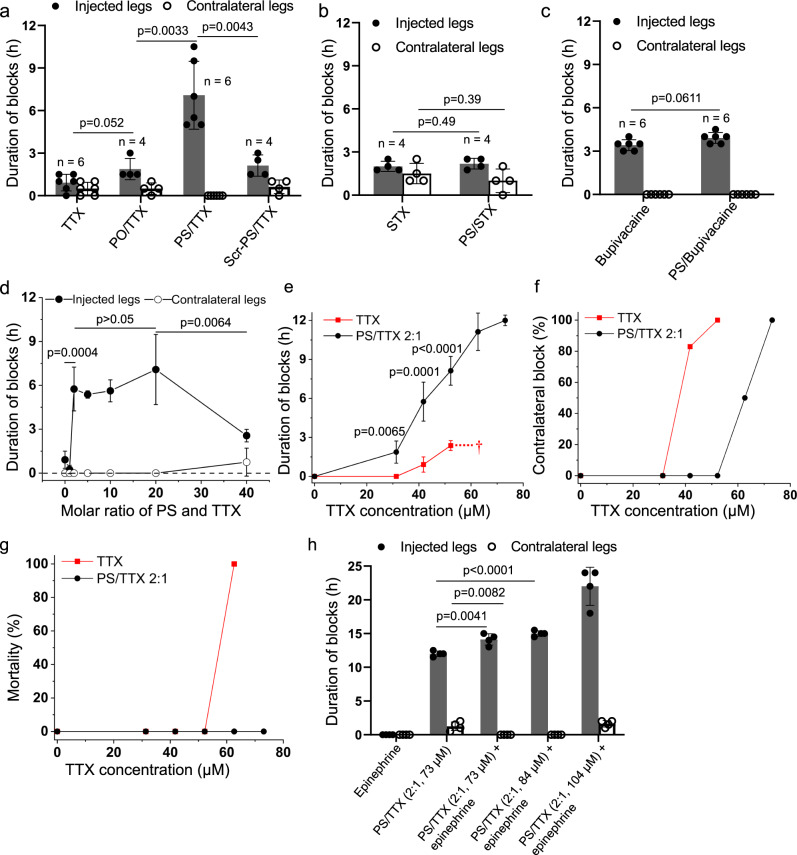
Peripheral nerve block with PS/TTX complexes. **a** Peripheral nerve blockade with 42 µM TTX, free or complexed with aptamers (PO, PS, Scr-PS) (*n* = 6 biologically independent animals for TTX and PS/TTX; *n* = 4 biologically independent animals for PO/TTX and Scr-PS/TTX). **b** Peripheral nerve blockade with 22 µM STX alone or combined with the TTX-specific PS aptamer (*n* = 4 biologically independent animals for STX and PS/STX). **c** Peripheral nerve blockade with bupivacaine alone or combined with the TTX-specific PS aptamer (*n* = 6 biologically independent animals for bupivacaine and PS/bupivacaine). The bupivacaine concentration in each formulation was 15.4 mM. **d** Peripheral nerve blockade with varying molar ratios of PS/TTX complexes, using 42 µM TTX. Data are means ± s.d. (*n* = 6 biologically independent animals for TTX and PS/TTX 20:1; *n* = 4 biologically independent animals for PS/TTX 1:1, 2:1, 5:1, 10:1, and 40:1). **e** Sciatic nerve blockade with free TTX and PS/TTX (2:1) in the injected hindpaws. The dagger indicates 100% mortality. Data are means ± s.d., *n* = 6 biologically independent animals for TTX 42 µM and TTX 52 μM; *n* = 4 biologically independent animals for TTX 31 μM, TTX 63 μM, and PS/TTX groups. **f** Frequency of nerve block in the contralateral (uninjected) hindpaws (*n* = 6 biologically independent animals for TTX 42 µM and TTX 52 μM; *n* = 4 biologically independent animals for TTX 31 μM and PS/TTX groups). **g** Animal mortality after treatment with TTX and PS/TTX (2:1) (*n* = 6 biologically independent animals for TTX 42 µM and TTX 52 μM; *n* = 4 biologically independent animals for TTX 31 μM, TTX 63 μM, and PS/TTX groups). **h** Effect of 55 µM epinephrine on the duration of sensory nerve blockade from PS/TTX (2:1, 73 µM, 84 µM and 104 µM TTX). Data are means ± s.d., *n* = 4 rats per group. Statistical comparisons were performed using Student t-test (two-sided). Source data are provided as a Source Data file.

Nerve block with 42 µM free TTX lasted 0.9 ± 0.6 h. Delivery as PO/TTX doubled block duration to 1.9 ± 0.8 h, and as PS/TTX increased it 7.7-fold, to 7.1 ± 2.4 h (Fig. 2a and Supplementary Fig. 4). Block from PS/TTX was statistically significantly longer than that from TTX (*p* = 0.0001) or PO/TTX (*p* = 0.0033).

To assess the possibility that nerve blocks were resulting from an inherent aptamer property (rather than from TTX), rats were injected at the left sciatic nerve with PO or PS aptamers (83.6 µM or 836 µM) without TTX. No nerve block was detected over a 6 h period (Supplementary Fig. 5 and Supplementary Table 2).

### Specificity of PS aptamer/TTX in vivo

To determine the specificity of the TTX-binding PS aptamer, three more control groups were utilized to verify the selectivity of the exact aptamer to TTX in vivo. First, the non-selective scrambled PS aptamer mentioned above was combined with TTX (Scr-PS/TTX) at a fixed molar ratio of 20:1. The Scr-PS/TTX (42 µM TTX) showed a 2.3-fold improvement of nerve block duration over free TTX (to 2.1 ± 0.8 h, *p* = 0.0207) which is likely due to non-specific interactions between Scr-PS and TTX, but far lower than that of the more selective PS/TTX moiety (7.7-fold, 7.1 ± 2.4 h, *p* = 0.0043) (Fig. 2a and Supplementary Fig. 4). Of note, both PS/TTX (20:1) and Scr-PS/TTX (20:1) had a very similar and low viscosity (<0.002 Pa s) determined by rheometry (Supplementary Fig. 6) which is not responsible for the differences of durations of nerve block. Second, the selectivity of the PS aptamer for TTX was confirmed by combining it with another S1SCB, STX which acts at the same site on the sodium channel as TTX^33^, has a similar molecular weight and guanidinium group but is otherwise structurally very different (Fig. 1c)^34^. 2 μg (22 μM) STX provided sensory nerve blockade lasting 2.0 ± 0.4 h, while nerve block from the same concentration of STX in a 20:1 molar ratio with the TTX-specific-PS (PS/STX) was unchanged at 2.2 ± 0.4 h (*p* = 0.49), showing that the TTX-binding aptamer did not prolong nerve block from STX (Fig. 2b). Third, an amino-amide local anesthetic, bupivacaine, was combined with the TTX-binding aptamer. It binds to the same sodium channel as TTX but at a different site on the inner surface of the cell membrane^35^, and is structurally very different (Fig. 1c). The combination of 836 μM PS aptamer with 15.4 mM bupivacaine hydrochloride did not significantly prolong the duration of nerve block (3.9 ± 0.4 h with PS/bupivacaine versus 3.4 ± 0.4 h from free bupivacaine; *p* = 0.0611) (Fig. 2c). These results confirm specific interactions between TTX and the TTX-binding PS aptamer.

### Optimization of the ratio of PS aptamer and TTX to enhance nerve blockade

We investigated the effect of the PS/TTX ratio (1:1, 2:1, 5:1, 10:1, 20:1, and 40:1) on the duration of nerve block, at a constant TTX concentration of 42 µM. These formulations were injected at the sciatic nerve site. PS/TTX with a molar ratio of 1:1 induced sensory nerve block lasting 0.3 ± 0.3 h, which was similar to the duration of block from the same concentration of free TTX (Fig. 2d). In the range of PS/TTX ratios from 2:1 to 20:1, block was markedly prolonged: 5.8 ± 1.5 h (2:1), 5.4 ± 0.3 h (5:1), 5.6 ± 0.8 h (10:1), 7.1 ± 2.4 h (20:1). There was no statistically significant difference in the observed injected or contralateral latencies over that range of molar ratios. However, when the PS/TTX ratio was increased to 40:1, the duration of nerve block was reduced to 2.6 ± 0.4 h. Consequently, PS/TTX with the molar ratio 2:1, which provided long nerve block time at the lowest aptamer concentration, was selected for subsequent studies.

All PS/TTX complexes had a very low viscosity less than 0.003 Pa s in the range of angular frequencies tested (Supplementary Fig. 7). There was no difference in the measured viscosity of the complexes at different ratios (*n* = 3, *p* > 0.05 for all comparisons), so differences in viscosity did not cause the differences in nerve block.

### Dose response with the optimized aptamer/TTX ratio

Dose-response experiments were performed in rat left sciatic nerve block model (*n* = 4 or 6) with free TTX drug or PS/TTX (2:1) complexes. Nerve blocks on the injected side were much longer with PS/TTX than with free TTX at all dosages used (Fig. 2e and Supplementary Table 2). The duration of nerve blockade increased with increasing PS/TTX dose (1.9 ± 0.9 h at 31 μM TTX, 12.0 ± 0.4 h at 73 μM TTX). PS enhanced the performance of low doses of TTX: 31 μM (3 μg) of free TTX caused no nerve block, while the same dose in PS/TTX produced a median duration of sensory nerve block of 1.9 ± 0.9 h, with successful nerve block in 100% of animals. Additionally, PS/TTX improved the safety of TTX as evidenced by the absence or reduction of systemic toxicity from TTX (i.e., deficits in the contralateral extremities; Fig. 2f and Supplementary Table 2). For example, 63 μM (6 μg) of free TTX was uniformly fatal, while no rats died at any dose of PS/TTX tested (Fig. 2g and Supplementary Table 2). Contralateral latency was dose-dependent with both TTX and PS/TTX, but it was much greater with TTX than PS/TTX. Motor blocks were 1.3-fold longer than sensory blocks for PS/TTX-treated rats (*p* < 0.05) (Supplementary Fig. 8).

### Potentiating local anesthetic activity of PS/TTX by addition of epinephrine

The prolongation of nerve block by PS is due to control of TTX release from the site of injection. This control allowed the delivery of doses of TTX that would otherwise be fatal. We postulated that the dose delivered—and therefore the duration of effect—could be further increased by co-injection of an agent that could slow release from the site of injection by pharmacological means, epinephrine^30,36,37^. Co-injection of PS/TTX (2:1) and 55 μM epinephrine at the sciatic nerve allowed TTX concentrations as high as 104 µM to be delivered without mortality (Fig. 2h). This is a dose where, in the absence of aptamer, TTX is uniformly fatal, and even with epinephrine administration there was a 67% fatality rate^30^. A duration of block of 22.0 ± 2.8 h could be achieved with 104 µM PS/TTX with epinephrine, almost twice the longest duration that could be achieved with PS/TTX without epinephrine and without animal mortality. Peak contralateral latency was markedly reduced by epinephrine, e.g., 12 s for 73 µM in PS/TTX and 2 s for PS/TTX with epinephrine (*p* < 0.0001) (Supplementary Fig. 9).

### Tissue retention of PS/TTX

**Fig. 3:**
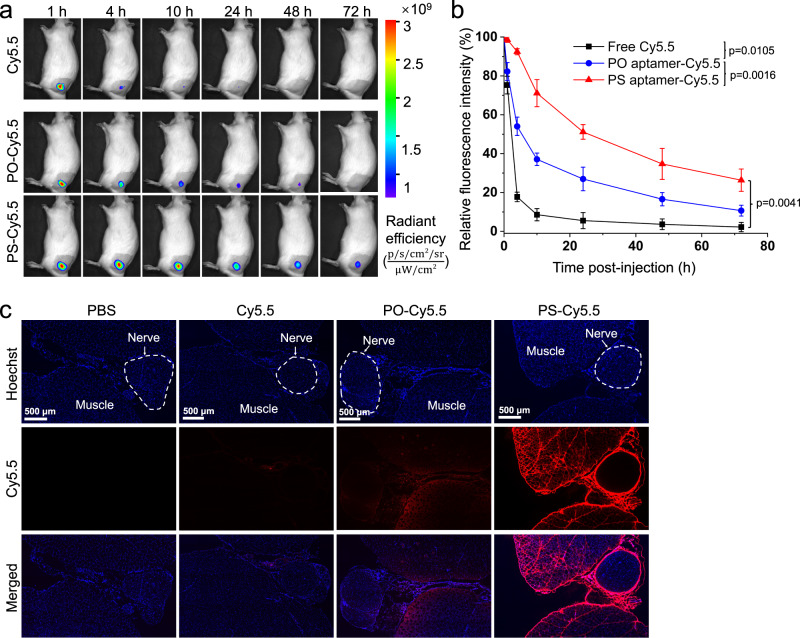
Tissue distribution of PS aptamer. **a** Representative time courses for the retention of Cy5.5-labelled aptamers (PO or PS) or free Cy5.5 at the site of injection as monitored by an IVIS. Color represents fluorescence from Cy5.5. **b** Quantification of the fluorescence intensity over time (as a percentage of intensity at time = 0, immediately after sciatic nerve injection), derived from data in panel (**a**). Data are means ± s.d., *n* = 4 rats per group. Statistical comparisons were performed using Student *t*-test (two-sided). **c** Representative confocal images of the rat sciatic nerve and surrounding tissue cryosections 4 h after sciatic nerve injection of Cy5.5 or Cy5.5-labelled aptamers (PO and PS). Red: Cy5.5, indicating formulations; blue: Hoechst33342, indicating cell nucleus. Each experiment was repeated three times independently with similar results. Source data are provided as a Source Data file.

To assess the local retention of aptamers in tissue, rats were injected at the left sciatic nerve with PO and PS aptamers covalently modified with the near-infrared fluorescence dye Cy5.5 at their 5’ end (Cy5.5-PO/PS, Supplementary Table 1). The fluorescence intensity at the injection site was monitored at predetermined intervals with an in vivo imaging system (IVIS). Free Cy5.5 was cleared rapidly with less than 20% left at 4 h, while approximately 63% of the PO aptamer was cleared within 10 h (Fig. 3a). With PS aptamer, ~50% of the peak fluorescence remained at 24 h. The fluorescence intensity of PS-Cy5.5 was statistically significantly stronger than for Cy5.5 and PO-Cy5.5 at all time-points tested (Fig. 3b). These data confirm that the PS aptamer allowed for longer tissue retention.

We harvested the sciatic nerves and surrounding tissues 4 h post-injection, processed them for histology, stained them with Hoechst 33342 (staining the nuclei), and imaged them by laser scanning confocal microscopy. Fluorescence from Cy5.5 was detected in the tissues surrounding the sciatic nerve in PS aptamer-injected rats that was ~4-fold higher than that in animals injected with the PO aptamer (Fig. 3c and Supplementary Fig. 10). Aptamer fluorescence was observed in the connective tissue between muscle and nerve (i.e., at the site of injection near the nerve), but not within the sciatic nerve itself (Fig. 3c and Supplementary Fig. 11).

The presence of TTX in tissue at pharmacologically relevant concentrations is revealed by the neurobehavioral bioassay: while nerve block persists, TTX is present; when nerve block resolves, the TTX/STX is essentially gone.

### Tissue reaction

**Fig. 4:**
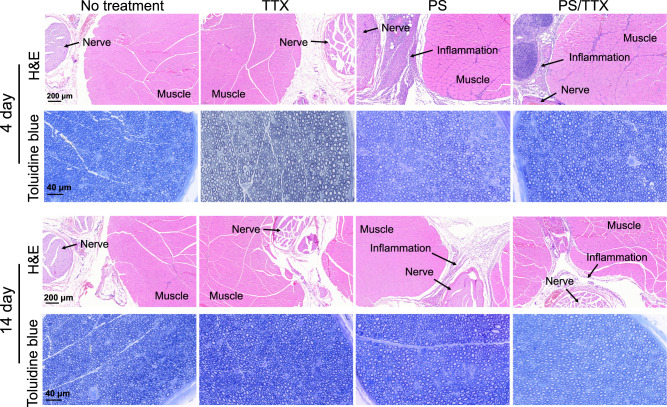
Tissue reaction to free TTX, PS aptamer, and PS/TTX complexes. H&E: Representative hematoxylin–eosin stained sections of muscles and adjacent loose connective tissue 4 and 14 days after sciatic nerve injection of TTX (52 μM), PS aptamer (146 μM), or PS/TTX (2:1, 73 μM TTX) in 0.3 mL of PBS. Toluidine Blue: Representative toluidine blue stained sections of nerve 4 days and 14 days after sciatic nerve injection of the above formulations. n = 4 in each group.

The sciatic nerve with surrounding tissues were harvested at days 4 and 14 after sciatic nerve injection and processed for histology (*n* = 4 for all groups; Fig. 4; Supplementary Table 3). Muscle tissue was stained with hematoxylin-eosin (H&E). Free TTX induced very mild inflammation (score = 0 or 1 in all animals), which is consistent with previous reports^38^. A mixed inflammatory response (score = 2 or 3 in all animals) was observed at the site of injection for the PS aptamer and PS/TTX groups at day 4. Inflammation diminished (score = 1 or 2 in all animals) by day 14 (*n* = 4, *p* < 0.05 compared to day 4). There was no myotoxicity in any treatment groups (score = 0) or untreated rats at any time point (Supplementary Table 3). To examine neurotoxicity, the sciatic nerves were embedded in Epon and stained with toluidine blue (H&E staining is relatively insensitive for identifying nerve injury). The PS aptamer and PS/TTX complexes did not cause any nerve injury at either time point (Fig. 4).

### In vivo nerve block of STX-binding PS aptamer (PSAP_STX_)/STX complexes

**Fig. 5:**
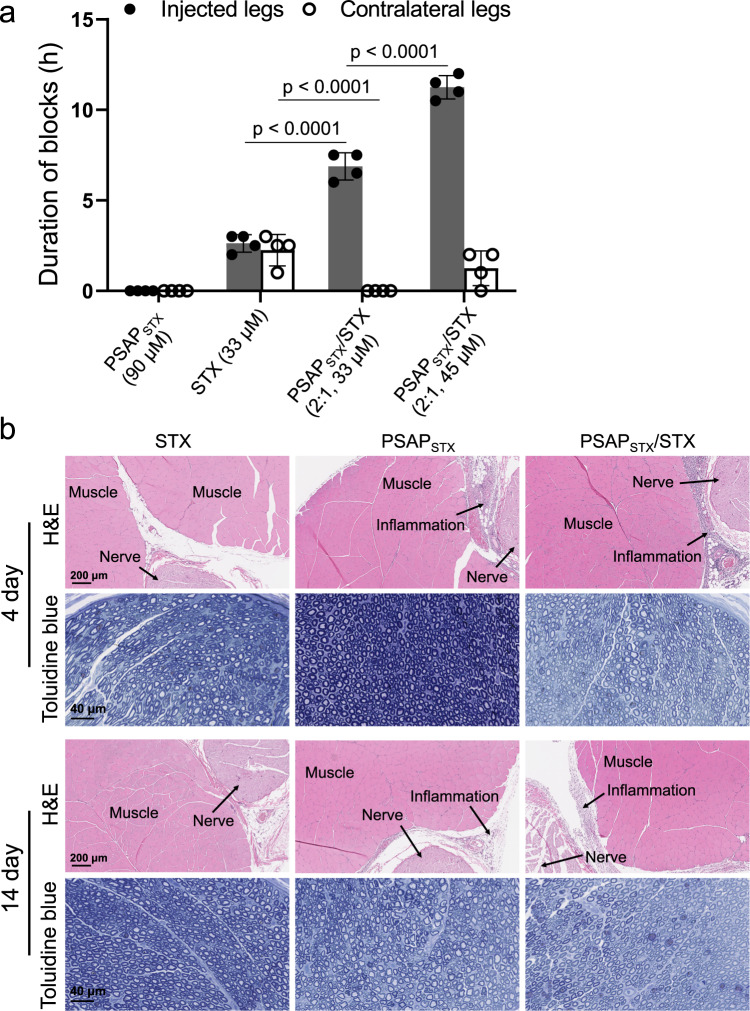
Peripheral nerve blockade effect of STX-binding PS aptamer (PSAPSTX)/STX. **a** Duration of sensory nerve blocks in the injected legs and uninjected (contralateral) extremities after sciatic nerve injection (*n* = 4 biologically independent animals). Data are means ± s.d. Statistical comparisons were performed using Student *t*-test (two-sided). **b** Tissue reaction to STX (33 μM), PSAP_STX_ (90 μM), and PSAP_STX_/STX (2:1, 45 μM STX) complexes 4 and 14 days after administration. Source data are provided as a Source Data file.

To test the generality of the use of aptamers as drug delivery systems, we investigated peripheral nerve block with STX with a previously reported PS aptamer specific to STX (PSAP_STX_, Supplementary Table 1)^39^. STX has approximately twice the potency of TTX in vivo for rat sciatic nerve block^40^. Injection of 33 µM free STX in PBS caused nerve block lasting 2.6 ± 0.5 h (Fig. 5a and Supplementary Table 4), with contralateral deficits in all animals (with a duration of 2.3 ± 0.9 h). In contrast, 33 µM STX in PSAP_STX_/STX (molar ratio 2:1) increased nerve block in the injected extremity 2.6-fold, to 6.9 ± 0.8 h (*p* < 0.0001), and the animals had no increase in contralateral latency (Fig. 5a). PSAP_STX_/STX (2:1) with 45 μM STX provided sensory nerve blockade lasting 11.3 ± 0.7 h and while there were increases in contralateral latency, no animal died, whereas injection of free STX at the same dose was uniformly fatal.

PSAP_STX_ and PSAP_STX_/STX had no cytotoxicity in C2C12 and PC12 cell lines by MTS assay (Supplementary Fig. S12). To assess tissue reaction, rats were euthanized 4 and 14 days after injection (*n* = 4 at each time point). The sciatic nerve and surrounding tissues were harvested and processed for histology by H&E staining (for inflammation and myotoxicity) or toluidine blue staining (for neurotoxicity). Inflammation was similar to what was seen with PS/TTX. Similarly, there was no myotoxicity or neurotoxicity in any animals treated with free PSAP_STX_ or PSAP_STX_/STX complexes at days 4 and 14 (Fig. 5b and Supplementary Table 5).

## Discussion

The present work demonstrates the use of the drug-specificity of aptamers to create highly effective depot-type drug delivery systems. Control of drug release was achieved by forming aptamer/drug non-covalent complexes, and in vivo results demonstrated that the efficacy and therapeutic index of small-molecular drugs (TTX and STX) had been enhanced remarkably in a rat sciatic nerve block model.

This approach was effective with two molecules that are of a class that is difficult to encapsulate in many traditional drug delivery systems: small, hydrophilic molecules. Moreover, incorporation of such molecules in many traditional drug delivery systems may result in marked burst release, potentially causing toxicity^31^. A number of approaches have been developed to improve their incorporation^41,42^, relying on approaches such as electrostatic interactions with charged compounds^43^, or covalent tethering to a polymer^20^. These approaches vary considerably in the degree to which they are effective or complicated. The approach shown here is straightforward—assuming that one can make the aptamer and that it binds the drug effectively.

The aptamer DDS described here has several advantages over existing systems. Aptamers are easy to synthesize and chemically modify. These aptamers were synthesized through solid-phase technology and phosphoroamidite chemistry, which allows the addition of multiple functional moieties at designated positions. These synthetic techniques are simple, efficient, easy to scale-up and inexpensive. Importantly, the process of drug loading is simple one-step mixing, which has clear advantages over many others, which are often multi-step and have relatively low efficiencies.

The specific drug-aptamer interaction is the basis for the controlled release functionality of this system. Consequently, this approach could be used for a wide range of therapeutics, assuming that an aptamer could be developed by existing approaches. However, each aptamer-based drug delivery system would only be effective for the drug for which it is specific. Here, we used local anesthesia as the in vivo model in which to demonstrate effect and safety. However, this technology could also be used for treatment of many other types of diseases, at least—based on our results—where depot-type drug delivery systems are desirable, whether to local effect (as in this case), or systemic. In systemic administration (i.e., intravenous injection of drug-aptamer complexes), the use of the aptamer’s specific interaction to bind drug would likely mean that the aptamer could not be used to target specific tissues.

PS chemical modification improves the in vivo stability of aptamers against degradation by nucleases^44,45^. PS modification can increase the binding affinity between aptamer and drug^7^, improving drug release kinetics. We speculate that the enhanced affinity of the PS aptamer to TTX can be attributed to the changed electrostatic and hydrophobic interactions between them due to the oxygen-to-sulfur substitutions in the DNA backbone^46^. This was seen with TTX (Fig. 1d, e) and resulted in prolongation of effect and reduction of toxicity (Fig. 2e). PS aptamers also demonstrated much longer tissue retention than PO aptamers (Fig. 3). Two potential explanations are that the PS backbone confers aptamer resistance against nuclease digestion^25,26^, and that PS has a high propensity to bind various proteins nonspecifically in vivo, which enhances tissue retention^47^. It is not clear how much the enhanced tissue retention was beneficial in prolonging nerve block from TTX, since the timeframe of nerve block was much shorter than the time frame of tissue retention, but it could be important for drugs with longer durations of effect.

The marked reduction in duration of effect of the PS/TTX formulation at the 40:1 ratio was unexpected. It may be due to steric or other interference with binding of TTX at high aptamer concentrations, interactions between aptamers at high concentration, or some other unknown mechanism.

Tissue reaction to the PS aptamer was benign at the doses tested when used locally at the rat sciatic nerve. Moreover, there is a track-record of aptamers in clinical use^48–50^. Therefore, safety issues are unlikely to be an obstacle in translation of an aptamer-based drug delivery system.

In conclusion, we have provided proof-of-concept of the use of drug-specific DNA aptamers as drug delivery systems. This approach provides a simple and safe method of delivery, at least for small-molecule drugs.

## Methods

### Ethical statement

The research complies with all relevant ethical regulations and was approved by the Boston Children’s Hospital Animal Care and Use Committee and carried out following the protocol (20-07-4222 R) in accordance with the guidelines of the International Association for the Study of Pain.

### Materials

Phosphoramidites and supplies for aptamer synthesis were purchased from Glen Research Co., USA. Mouse C2C12 myoblast (CRL-1772) and rat PC12 pheochromocytoma (CRL-1721) cell lines were purchased from American Type Culture Collection (Rockville, MD, USA). Sulfo-Cyanine5.5 amine (Cy5.5, 95.0%) was acquired from Lumiprobe Corporation (Hunt Valley, Maryland, USA). Tetrodotoxin (TTX) was purchased from Abcam (Waltham, MA, USA). TTX ELISA kits were purchased from Reagen LLC (Moorestown, NJ, USA). Dulbecco’s minimum essential medium (DMEM), horse bovine serum (HBS), fetal bovine serum (FBS), and Penicillin Streptomycin were purchased from Thermo Fisher Scientific Inc. (Waltham, MA, USA). STX was obtained from the U.S. Food and Drug Administration (FDA). Fluorescein sodium salt and phosphate buffered saline (PBS) were purchased from Sigma-Aldrich Co. (MO, USA). MALDI-TOF MS measurements were performed on a Bruker Microflex LT mass spectrometer (Bruker Daltonics Inc., MA, USA). Reverse-phase HPLC was carried out at a Waters (Waters Co., MA, USA) Breeze 2 HPLC system coupled to a SunFire C18 5 μm, 10 × 100 mm reverse phase column and a 2998 PDA detector, using TEAA buffer (0.1 M) and HPLC-grade acetonitrile as mobile phases. DLS data were recorded on Malvern Zetasizer Pro. Histology studies were carried out at the Kock Institute Swanson Biotechnology Center of Massachusetts Institute of Technology and at iHisto Inc.

### Aptamer synthesis

The synthesis of DNA aptamers (both phosphodiester [PO] and phosphorothioate [PS] versions) was performed on a Model 391 DNA synthesizer (Applied Biosystems, Inc., CA, USA) using standard solid phase phosphoramidite methodology. Deprotection of aptamers was carried out with ammonium hydroxide (28% NH_3_ in H_2_O) for 24 h at room temperature. The crude products were purified by reverse-phase HPLC liquid chromatography. Subsequently, the purified aptamers were treated with 20% acetic acid in H_2_O for 1 h to remove the dimethoxytrityl (DMT) protecting group, followed by extraction with ethyl acetate three times in an aqueous solution. The resultant aptamers were quantified using NanoDrop™ OneC microvolume UV-vis spectrophotometer and stored at −20 °C after lyophilization. To synthesize the dye-labelled aptamer, cyanine 5 (Cy5) or cyanine 5.5 (Cy5.5) was incorporated at the 5’-terminus by using Cy 5 phosphoramidites and Cy 5.5 phosphoramidites, respectively. The successful syntheses of all aptamers in this work were confirmed by MALDI-TOF MS.

### Preparation of aptamer/TTX and aptamer/STX complexes

Briefly, the TTX-binding aptamers (PO or PS) were dissolved in 1 × PBS (154 mM NaCl, 5.6 mM Na_2_HPO_4_, 1.1 mM KH_2_PO_4_; pH 7.4) in microcentrifuge tubes and heated to 95 °C for 5 min, followed by slow cooling to room temperature. After the annealing process, the TTX, dissolved in citrate buffer (5 mg/mL), was diluted with PBS (200 μg/mL) and then added to the aptamer solution at a predetermined molar ratio. The resulting solution was gently shaken overnight at room temperature for further tests. Similarly, the aptamer/STX complexes were prepared using the identical method.

### Microscale thermophoresis (MST) analysis of binding interactions

The immobilization-free MST is a rapid and precise method to study the small molecule-aptamer interactions in solution. It monitors the thermophoretic movement of different molecular ratios between target molecule and ligand through µm-sized temperature gradients. To establish these ratios, a constant amount of Cy5-labelled aptamer is mixed with different amounts of ligand. A serial dilution of TTX in the PBS was carried out to provide solutions having a range of concentrations between 152.6 nM and 5 mM. 5 µl of each solution was mixed with 5 µl of the Cy5-labelled aptamer, which was held at a constant concentration of 16 nM. The final concentrations of TTX in each capillary ranged from 76.3 nM to 2.5 mM. Each sample was analyzed on a Monolith NT. Automated (NanoTemper Technologies, Munich, Germany) at 25 °C, with 40% LED power and 80% laser power. Data were fitted using MO. Affinity Analysis software (version 2.3, NanoTemper Technologies) and MST-on time was set at 1.5 s to determine the aptamer K_d_ values.

### In vitro TTX release

The TTX release kinetics from aptamer/TTX complexes was determined by placing 200 µL of these complexes (TTX, 42 µM) into a Slide-A-Lyzer MINI dialysis device with a 3500 MW cut-off, further dialyzed with 14 mL PBS and incubated at 37 °C on a platform shaker. At predetermined intervals, the dialysis solution was exchanged with fresh, pre-warmed PBS. The concentration of released TTX in media was quantified by an enzyme-linked immunosorbent assay (ELISA).

### Determination of viscosity

The rheological properties of aptamers and aptamer/TTX were measured using an AR2000 rheometer (TA Instruments, New Castle, DE, USA) with parallel plate geometry and a temperature controller. A parallel plate with a diameter of 20 mm was used, between which the gap distance was set as 0.3 mm. The dynamic properties were followed as a function of time at a constant shear stress of 0.1 Pa and an oscillation frequency of 0.01 rads^−1^, in frequency sweep tests (in the frequency ranging from 0.01 to 100 rads^–1^) at room temperature.

### Cell culture

C2C12 mouse myoblasts were cultured in Dulbecco’s modified Eagle’s medium (DMEM) supplemented with 20% fetal bovine serum (FBS) and 1% Penicillin Streptomycin. To induce differentiation into myotubes, C2C12 cells (8.0 × 10^3^) were seeded into 24-well plates and incubated in DMEM with 2% horse serum and 1% Penicillin Streptomycin for 7-10 days. The differentiation media was exchanged every 2 to 3 days. PC12 rat adrenal gland pheochromocytoma cells were grown in DMEM with 5% FBS, 5% horse serum, and 1% Penicillin Streptomycin. For neuronal induction, PC12 cells were seeded in 24-well plates at a density of 1.0 × 10^4^ cells per well and cultured in DMEM with 5% FBS, 5% horse serum, and 50 ng/mL nerve growth factor (NGF) for 10-14 days.

### MTS cytotoxicity assay

The cytotoxicity of aptamers, TTX, and aptamer/TTX complexes was evaluated with the MTS colorimetric assay. The C2C12 and PC12 cells were treated with varying doses of free TTX, aptamers, or the aptamer/TTX complexes. Cells incubated with vehicle (PBS) were set as a control. After 24 h of incubation, 40 μL of the MTS-based CellTiter 96® AQ_ueous_ One Solution Reagent was added to each well. The cells were incubated for another 4 h, and the absorbances (490 nm) were measured on a BioTek® Synergy™ Mx microplate reader (BioTek Inc., VT, USA).

### Animal studies

Animal studies were approved by the Boston Children’s Hospital Animal Care and Use Committee and carried out following protocols in accordance with the guidelines of the International Association for the Study of Pain. Adult male Sprague-Dawley rats (Charles River Laboratories, Wilmington, MA, USA) weighing 400–500 g were housed in groups under a 12-h/12-h light/dark cycle with lights on at 6:00 AM.

### Sciatic nerve block and neurobehavioral testing

The rats were randomly assigned to each group and injected with 300 μL of each formulation at the left sciatic nerve under brief isoflurane-oxygen anesthesia. A 23 G needle was introduced postero-medial to the greater trochanter, pointing in the anteromedial direction. Upon touching the bone, the drugs were injected onto the sciatic nerve. Then the rats underwent neurobehavioral testing at predetermined intervals.

The sensory nerve block was assessed using a modified hotplate test as described previously^51^. Briefly, the plantar surface of the rat’s hind paw was placed on a preheated hot plate (Model 39D Hot Plate Analgesia Meter; IITC) at 56 °C. The time was recorded with a stopwatch when the rat withdrew its foot (the thermal latency). The paw was removed from the hot plate to avoid injury if the rat did not retract the foot after 12 s. This test was repeated three times (with a 10 s pause between tests) at each time interval. A thermal latency above 7 s indicated a successful nerve blockade for the purpose of calculating the duration of the nerve block.

The motor nerve block was evaluated using a weight-bearing test in which the motor strength of the rat’s hindpaw was determined, as reported previously. Briefly, the rat was positioned with one hindpaw on a digital balance and was allowed to bear its own weight. The maximum weight that it could bear without the ankle touching the balance was recorded. The duration of motor blockade was defined as the time for weight-bearing to return halfway to normal from the maximal block as described previously^30^.

### In vivo imaging system (IVIS) imaging

The Sprague-Dawley rats were partly shaved and injected with 0.3 mL of free Cy5.5 or Cy5.5-labelled aptamers (Cy5.5, 4.64 μM) at the left sciatic nerve under the isoflurane-oxygen anesthesia. They were scanned at 0, 1, 4, 10, 24, 48, and 72 h post-injection using an IVIS 200 imaging system (Caliper Life Sciences, Inc. MA, USA). Quantitative analysis will be performed using the Live Imaging software of the IVIS. The half-life of tissue retention is the time required for the fluorescence intensity to decrease by 50% after injection and was calculated based on the fluorescence intensity.

### Tracking the location of aptamers in tissue using confocal imaging

Rats were injected with Cy5.5 or Cy5.5-labelled aptamers (84 µM) in 0.3 mL PBS at the left sciatic nerve under the isoflurane-oxygen anesthesia. They were then euthanized with carbon dioxide at 4 h post-injection. Sciatic nerves and surrounding tissues were harvested, embedded into the OCT compound (Fisher Scientific Inc., USA) and stored at −20 °C. The frozen tissues were cut into 8 μm-thick sections using a cryostat microtome, which were mounted onto glass slides. The slides were then fixed with 4% paraformaldehyde, stained with Hoechst33342, and imaged on an LSM-880 confocal laser scanning microscopy (Carl Zeiss Ltd., Cambridge, UK).

### Histology

The Sprague-Dawley rats (*n* = 4) were treated with 0.3 mL of aptamers (146 μM), free TTX (52 μM), or aptamer/TTX (146 μM/73 μM) at the left sciatic nerve, and then euthanized to assess the acute (at day 4) and chronic (at day 14) inflammation and tissue injury respectively. The sciatic nerve and surrounding tissue were collected, fixed in 10% neutral buffered formalin, and underwent standard processing for H&E-stained slide production. The slides were analyzed and scored for the presence of inflammation (0–4) and myotoxicity (0–6) by an independent, board-certified pathologist (Matthew Gregory Torre) who was blinded to the nature of the individual samples. The inflammation score is a subjective quantification of severity in which 0 was normal and 4 was severe inflammation (0: no inflammation, 1: peripheral inflammation, 2: deep inflammation, 3: muscular hemifascicular inflammation, 4: muscular holofascicular inflammation)^37^. The myotoxicity score is determined by the nuclear internalization and regeneration of myocytes^38^. Nuclear internalization is characterized by myocytes having nuclei located away from their usual location at the periphery of the cell. Regeneration is characterized by the presence of shrunken myocytes with basophilic cytoplasm. The scoring scale is as follows: 0 = normal; 1 = perifascicular internalization; 2 = deep internalization (more than five cell layers); 3 = perifascicular regeneration; 4 = deep tissue regeneration (more than five cell layers); 5 = hemifascicular regeneration; 6 = holofascicular regeneration.

To access the neurotoxicity of the aptamers’ formulations, the sciatic nerves were fixed in Karnovsky’s KII solution (1.25% formaldehyde, 2.5% glutaraldehyde, and 0.03% picric acid in 0.1 M sodium cacodylate buffer, pH 7.4.). The fixed tissues were washed with 0.1 M sodium cacodylate buffer and post-fixed with 1% osmium tetroxide/1.5% potassium ferrocyanide (in H_2_O) for 2 h. Samples were then washed in a maleate buffer and post-fixed in 1% uranyl acetate in maleate buffer for 1 h. Tissues were then rinsed in ddH_2_O and dehydrated through a series of ethanol (50%, 70%, 95%, (2x)100%) for 15 min per solution. Dehydrated tissues were put in propylene oxide for 5 min before they were infiltrated in epon mixed 1:1 with propylene oxide overnight at 4 °C. Samples were polymerized in a 60 °C oven in epon resin for 48 h. They were then sectioned into 500 nm thin sections, which were stained with toluidine blue and imaged on high-resolution light microscopy.

### Statistics and reproducibility

All quantitative measurements (i.e., TTX binding affinity analysis, TTX release, quantification of the fluorescence intensity) have at least three independent repeats. We calculated sample size in our studies by using a power analysis. Previous animal studies, or small pilot studies, when necessary, served as the basis for calculations of expected averages and deviations used to calculate power, for which we set experiment studies to a value of 0.8. This typically resulted in an experimental group size to be *n* = 4–6, depending on the experiment. Sample size is explicitly stated for each experimental group for individual experiments in figure captions and data descriptions. No data were excluded from the analyses. Origin 2022b and GraphPad Prism 9 were used for plotting. ImageJ (Version 1.53t) was used for image processing. MO. Affinity Analysis (version 2.3) software was used for binding affinity analysis. Statistical comparisons were performed using GraphPad Prism 9. Statistical comparisons were performed using Student *t*-test (two-sided) unless stated otherwise. Thermal latency, inflammation, and myotoxicity scores are reported as medians and quartiles due to their ordinal or non-Gaussian character. Data are presented as mean ± standard deviation. Statistical significance was set at *p* < 0.05.

### Reporting summary

Further information on research design is available in the Nature Portfolio Reporting Summary linked to this article.

## Supplementary information

Supplementary information

Reporting Summary

## Data Availability

The data that support the findings of this study are available within the article and its Supplementary Information files. Source data are provided as a Source Data file. Source data are provided with this paper.

## Source data

Source Data
