# Supplementary material for: An aptamer-based depot system for sustained release of small molecule therapeutics
Source: Nat Commun. 2023 Apr 28;14:2444. doi: 10.1038/s41467-023-37002-0 (PMC10147605; doi:10.1038/s41467-023-37002-0)
Supplement: Supplementary file 1 — Supplementary information [file 41467_2023_37002_MOESM1_ESM.pdf]

Supplementary information

An aptamer-based depot system for sustained release of small molecule therapeutics

Supplementary Tables

Supplementary Table 1. DNA aptamers sequences used in this study.

Strand description	Sequence
TTX-binding PO aptamer	5’-AAAAATTTCACACGGGTGCCTCGGCTGTCC-3’
Cy5.5-labeled TTX-binding PO aptamer	5’-Cy5.5-AAAAATTTCACACGGGTGCCTCGGCTGTCC-3’
Cy5-labeled TTX-binding PO aptamer	5’-Cy5-AAAAATTTCACACGGGTGCCTCGGCTGTCC-3’
TTX-binding PS aptamer	5’-A*A*A*A*A*T*T*T*C*A*C*A*C*G*G*G*T*G*C*C*T*C*G*G*C*T*G*T*C*C-3’
Cy5.5-labeled TTX-binding PS aptamer	5’-Cy5.5-A*A*A*A*A*T*T*T*C*A*C*A*C*G*G*G*T*G*C*C*T*C*G*G*C*T*G*T*C*C-3’
Cy5-labeled TTX-binding PS aptamer	5’- Cy5-A*A*A*A*A*T*T*T*C*A*C*A*C*G*G*G*T*G*C*C*T*C*G*G*C*T*G*T*C*C-3’
Scrambled PS aptamer	5’-G*G*C*T*C*C*A*C*T*C*A*T*A*C*T*T*C*C*G*A*G*T*A*A*G*A*C*G*G*T*-3’
Cy5-labeled scrambled PS aptamer	5’-Cy5-G*G*C*T*C*C*A*C*T*C*A*T*A*C*T*T*C*C*G*A*G*T*A*A*G*A*C*G*G*T*-3’
Complementary strand of TTX-binding PS aptamer	5’-G*G*A*C*A*G*C*C*G*A*G*G*C*A*C*C*C*G*T*G*T*G*A*A*A*T*T*T*T*T*-3’
STX-binding PS aptamer	5’-T*T*G*A*G*G*G*T*C*G*C*A*T*C*C*C*G*T*G*G*A*A*A*C*A*G*G*T*T*C*A*T*T*G-3’

Asterisk (*): phosphorothioate internucleotide linkage; PO: phosphodiester; PS: phosphorothioate; Scrambled PS aptamer: randomly rearranging the nucleotide sequence of TTX-binding PS aptamer

Supplementary Table 2. Efficacy of TTX or aptamer/TTX conjugates in sciatic nerve blockade.

Sample	TTX (μM)	Sensory Block time (h)	% Blocks injected legs	% Contralateral block	N	Mortality (%)
Free TTX	31	0	0	0	4	0
	42	0.9 ± 0.6	83	83	6	0
	52	2.4 ± 0.4	100	100	6	0
	63	-	-	-	4	100
PS/TTX 1:1	42	0.3 ± 0.3	50	0	4	0
PS/TTX 2:1	31	1.9 ± 0.9	100	0	4	0
	42	5.8 ± 1.5	100	0	4	0
	52	8.1 ± 1.1	100	0	4	0
	63	11.1 ± 1.4	100	50	4	0
	73	12 ± 0.4	100	100	4	0
PS/TTX 5:1	42	5.4 ± 0.3	100	0	4	0
PS/TTX 10:1	42	5.6 ± 0.8	100	0	4	0
PS/TTX 20:1	42	7.1 ± 2.4	100	0	6	0
PO/TTX 20:1	42	1.9 ± 0.8	100	75	4	0
Scr-PS/TTX 20:1	42	2.1 ± 0.8	100	75	4	0
PS/TTX 40:1	42	2.6 ± 0.4	100	0	4	0
PO aptamer	0	0	0	0	4	0
PS aptamer	0	0	0	0	4	0

Supplementary Table 3. Myotoxicity and inflammation for TTX formulations.

Sample	TTX (μM)	Aptamer (μM)	Myotoxicity score Day 4	Myotoxicity score Day 14	Inflammation score Day 4	Inflammation score Day 14
No treatment	0	0	0 (0-0)	0 (0-0)	0 (0-0)	0 (0-0)
Free TTX	52	0	0 (0-0)	0 (0-0)	0.5 (0-1)	0 (0-0)
P value			> 0.05	> 0.05	> 0.05	> 0.05
TTX-binding PS aptamer	73	146	0 (0-0)	0 (0-0)	2.5 (2-3)	1 (1-2)
P value			> 0.05	> 0.05	< 0.05	< 0.05
PS/TTX	73	146	0 (0-0)	0 (0-0)	3 (2-3)	1 (1-2)
P value			> 0.05	> 0.05	< 0.05	< 0.05

Inflammation scores: 0–4; myotoxicity scores: 0–6. Data are medians with 25th and 75th percentiles in parentheses. P values are for the comparison of the tissue reaction of test compounds to that of the untreated group; n = 4 rats for all groups. Statistical comparisons were performed using Student t-test (two-sided).

Supplementary Table 4. Nerve block in injected and contralateral hindpaws after injection of STX or STX-binding aptamer/STX conjugates.

Sample	STX (μM)	Sensory Block time (h)	% Blocks injected legs	% Contralateral block	N	Mortality (%)
STX	33	2.6 ± 0.5	100	100	4	0
	45	-	-	-	4	100
$PSAP_{STX}$/STX (2:1)	33	6.9 ± 0.8	100	0	4	0
	45	11.3 ± 0.6	100	75	4	0
$PSAP_{STX}$	90	0	0	0	4	0

Data are means ± s.d. $PSAP_{STX}$: PS aptamer specific to STX.

Supplementary Table 5. Myotoxicity and inflammation for STX formulations.

Sample	TTX (μM)	Aptamer (μM)	Myotoxicity score Day 4	Myotoxicity score Day 14	Inflammation score Day 4	Inflammation score Day 14
No treatment	0	0	0 (0-0)	0 (0-0)	0 (0-0)	0 (0-0)
STX	33	0	0 (0-0)	0 (0-0)	0.5 (0-1)	0 (0-0)
P value*			> 0.05	> 0.05	> 0.05	> 0.05
$PSAP_{STX}$	0	90	0 (0-0)	0 (0-0)	2.5 (2-3)	2 (2-2)
P value*			< 0.05	> 0.05	< 0.05	< 0.05
$PSAP_{STX}$/ STX	45	90	0 (0-0)	0 (0-0)	2 (2-3)	1 (1-2)
P value*			> 0.05	> 0.05	< 0.05	< 0.05

Inflammation scores: 0-4; myotoxicity scores: 0-6. Data are medians with 25th and 75th percentiles in parentheses. *P values are for the comparison of the tissue reaction of test compounds to that of the untreated group; n = 4 rats for all groups. Statistical comparisons were performed using Student t-test (two-sided).

Supplementary Figures

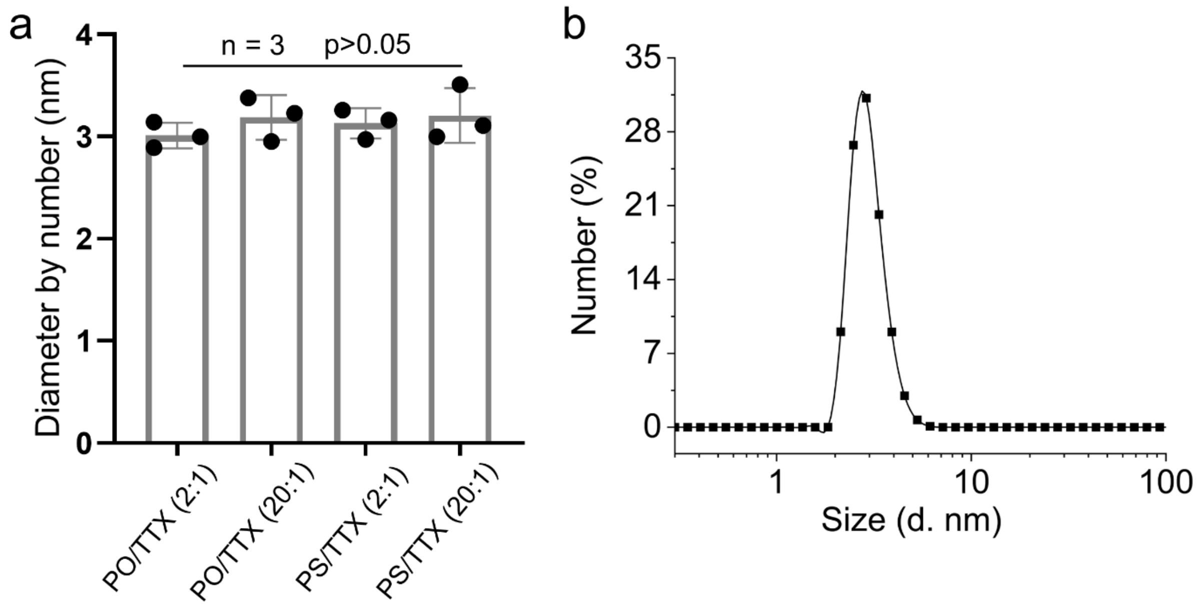

Supplementary Fig. 1 (**a**) The size of aptamer/TTX conjugates at different molar ratios of aptamers to TTX, as measured by dynamic light scattering (DLS). The TTX concentration was fixed (42 μM). Data are means ± s.d., n=3 independent experiments. Statistical comparisons were performed using Student t-test (two-sided). (**b**) Representative number-average size distribution of aptamer/TTX. Source data are provided as a Source Data file.

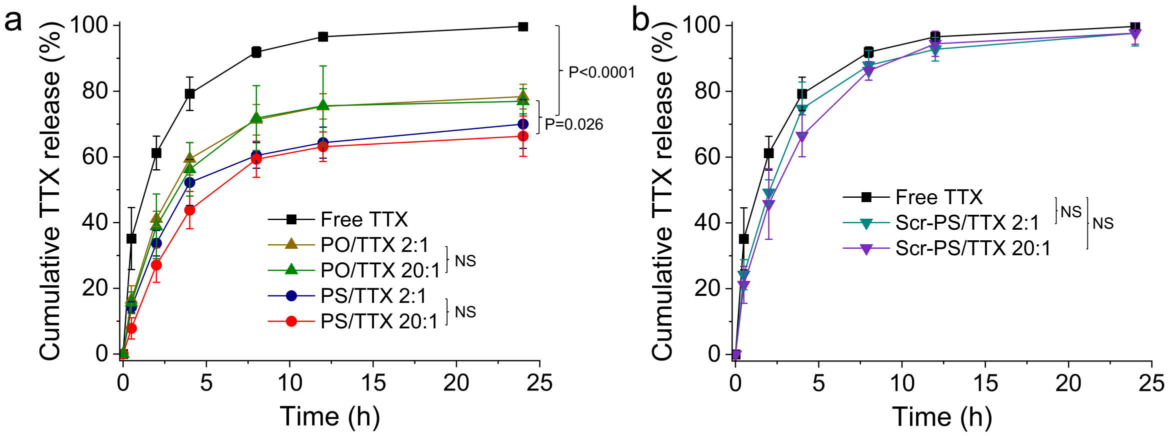

Supplementary Fig. 2 TTX release kinetics from aptamer/TTX conjugates and controls over 24 h. (**a**) Free TTX versus PO/TTX versus PS/TTX. (**b**) Free TTX versus Scr-PS/TTX. The TTX concentration for each group was 42 μM. Data are shown as the mean ± s.d., n = 4 independent experiments. Statistical comparisons were performed using Student t-test (two-sided). NS, not statistically significant. Source data are provided as a Source Data file.

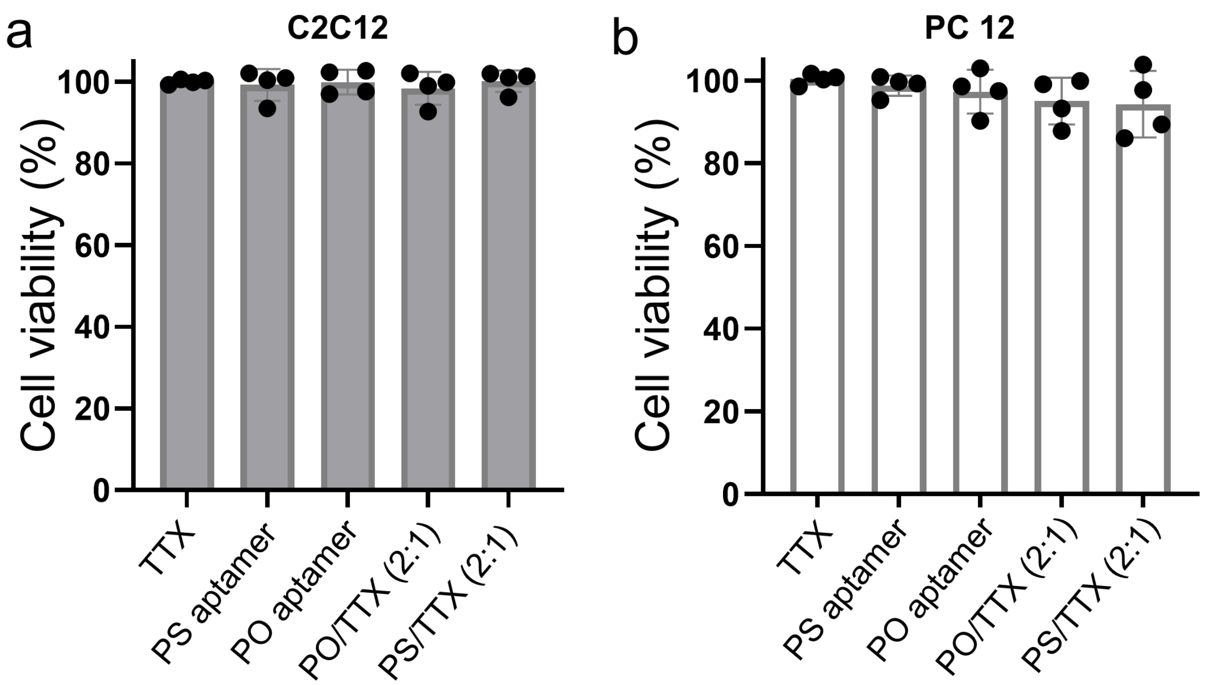

Supplementary Fig. 3 MTS cytotoxicity assay for (**a**) myotoxicity (in C2C12 cells) and (**b**) neurotoxicity (in PC12 cells) after 24 h exposure to the following groups: free TTX, PO aptamer, PS aptamer, PO/TTX (2:1), or PS/TTX (2:1). The TTX concentration was 73 µM and the aptamer concentration was 146 µM. Data are means ± s.d., n= 4 biological replicates per group. Source data are provided as a Source Data file.

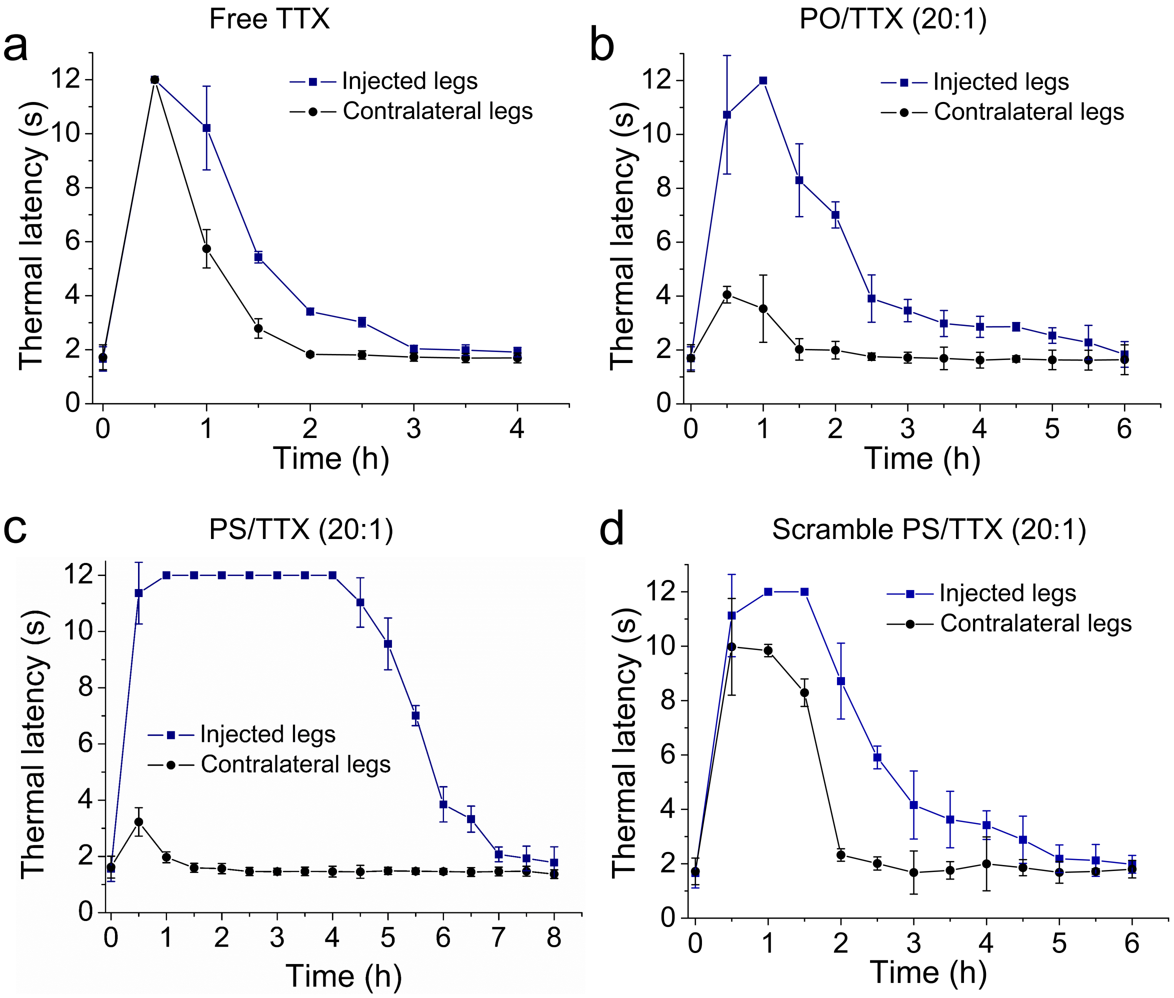

Supplementary Fig. 4 Representative time courses of thermal latency after sciatic nerve injections of aptamer formulations. Rats were injected with 0.3 mL of (**a**) free TTX, (**b**) PO/TTX (20:1), (**c**) PS/TTX (20:1), or (**d**) scrambled PS/TTX (20:1) in PBS, all with 42 µM TTX. Data are means ± s.d., n = 4 biologically independent animals per group. Source data are provided as a Source Data file.

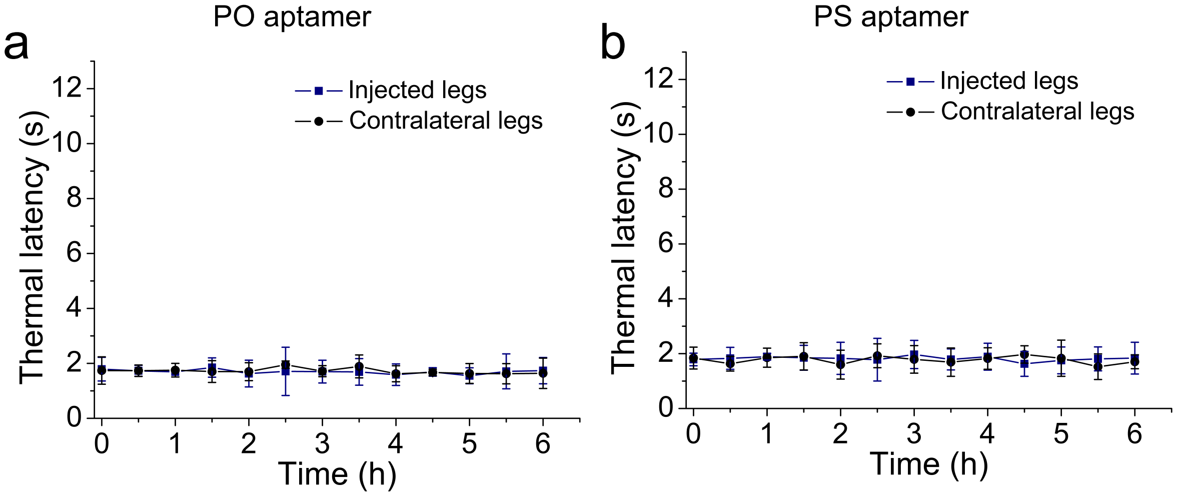

Supplementary Fig. 5 Representative time courses of thermal latency after sciatic nerve injections of (**a**) PO aptamer or (**b**) PS aptamer. All injections were in 0.3 mL of PBS at an aptamer concentration of 836 μM. Data are means ± s.d., n = 4 biologically independent animals per group. Source data are provided as a Source Data file.

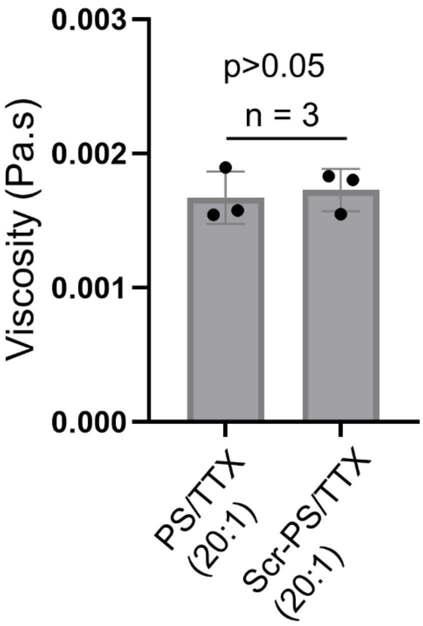

Supplementary Fig. 6 Viscosities of the PS/TTX (20:1) and Scr-PS/TTX (20:1). The concentration of TTX was 42 μM. There was no statistically significant difference between these two groups (n=3, $p>0.05$). Data are means ± s.d., n=3 independent experiments. Statistical comparisons were performed using Student t-test (two-sided). Source data are provided as a Source Data file.

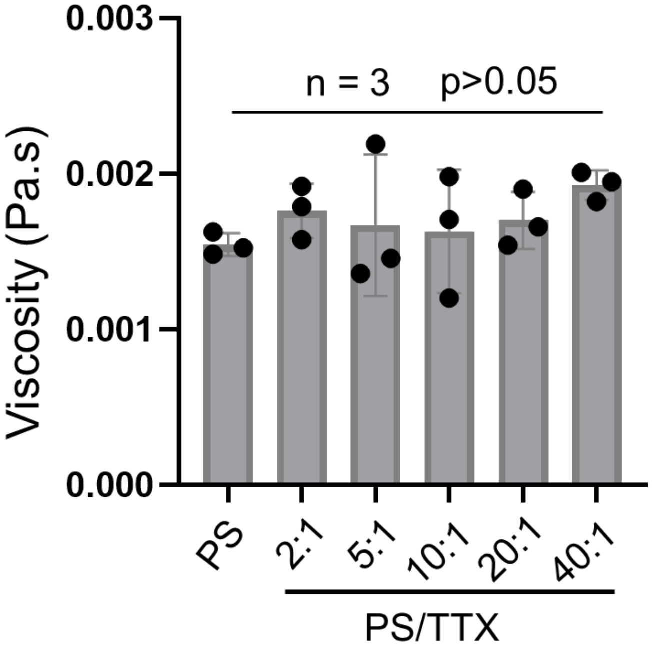

Supplementary Fig. 7 Viscosities of the PS aptamer alone (84 μM) and differing molar ratios of PS aptamer to TTX. The concentration of TTX was 42 μM. There was no difference in the measured viscosity of the various ratios (n=3, all $p>0.05$ for all comparisons). Data are means ± s.d., n=3 independent experiments. Statistical comparisons were performed using Student t-test (two-sided). Source data are provided as a Source Data file.

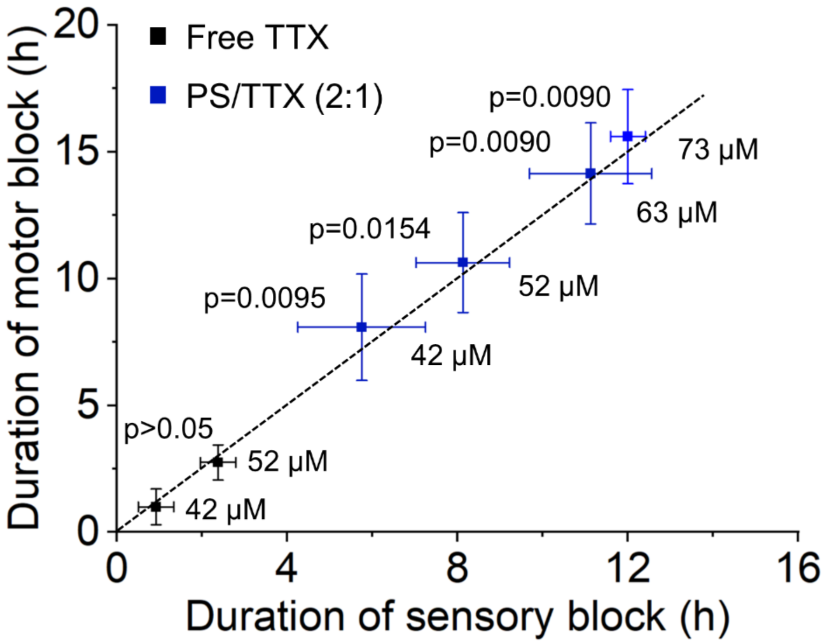

Supplementary Fig. 8. Representative comparison of the duration of sensory and motor blocks. The rats were injected with free TTX or PS/TTX (2:1) in 0.3 mL of PBS. The TTX concentration for each group has been indicated in the figure. P values are for the comparison of the duration of sensory block of formulations to that of motor block. Data are means ± s.d.; n = 4 biologically independent animals per group. Statistical comparisons were performed using Student t-test (two-sided). Source data are provided as a Source Data file.

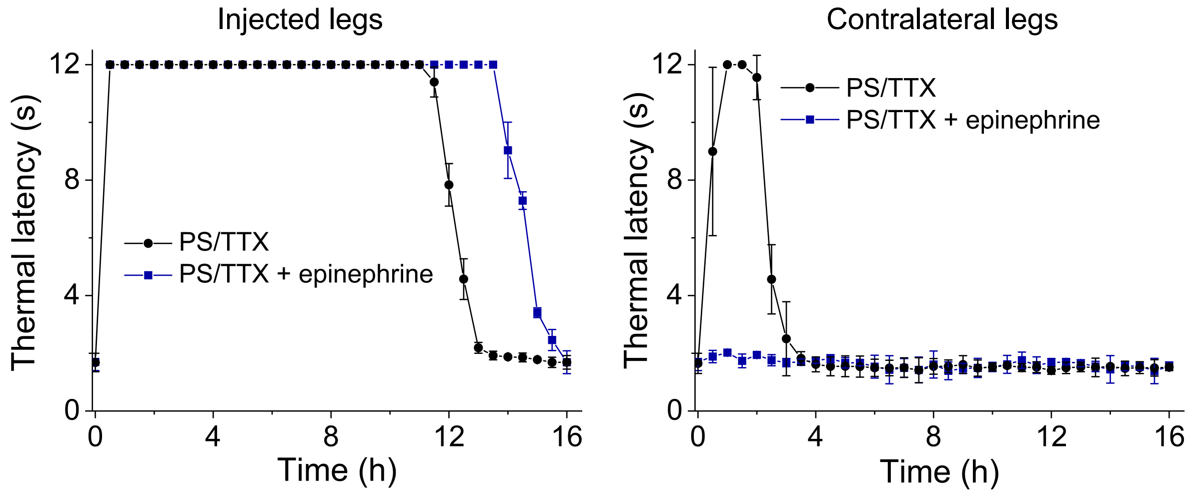

Supplementary Fig. 9 Representative time courses of thermal latency after sciatic nerve injections of PS/TTX (2:1, 73 μM TTX) or in combination with 55 μM epinephrine. Data are means ± s.d.; n = 4 biologically independent animals per group. Source data are provided as a Source Data file.

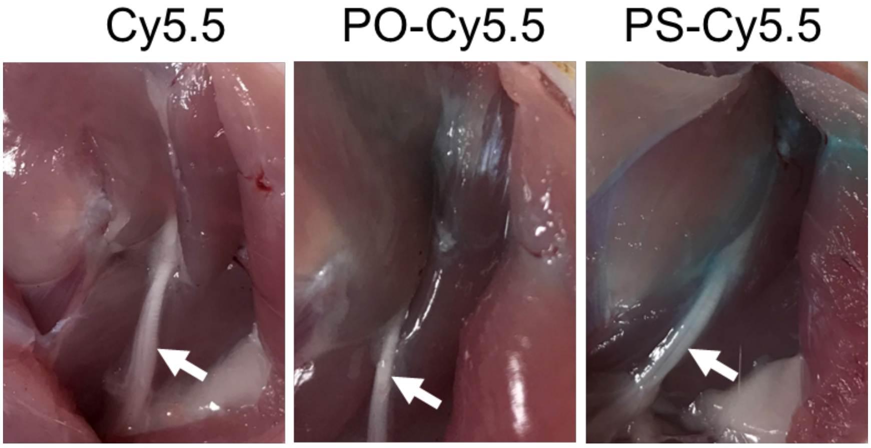

Supplementary Fig. 10. Representative photographs of dissected sciatic nerve (white arrow) and surrounding tissues of rats 4 hours after sciatic nerve injection. The blue color is from Cy5.5.

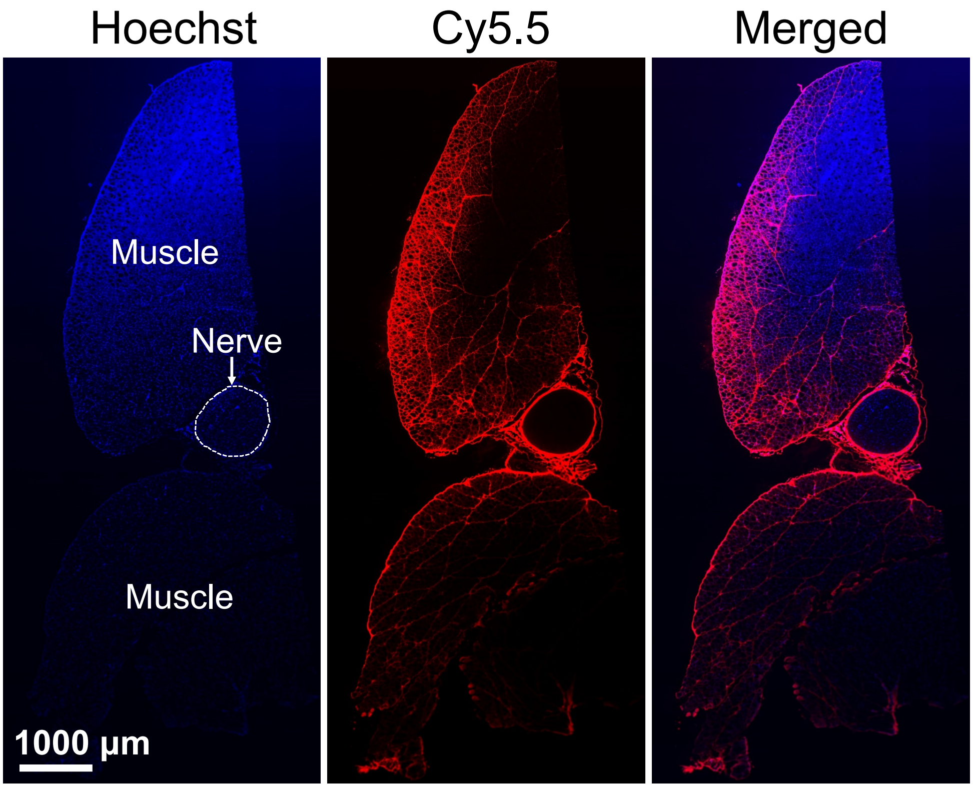

Supplementary Fig. 11. Confocal images of the rat sciatic nerve and surrounding tissue cryosections 4 h after sciatic nerve injection of Cy5.5-labelled PS aptamer. Red: Cy5.5, indicating aptamer; blue: Hoechst 33342, indicating nuclei. Each experiment was repeated three times independently with similar results.

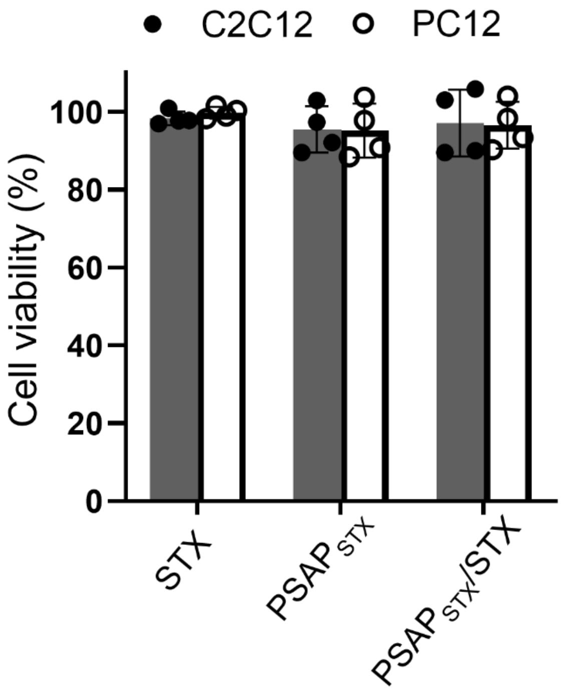

Supplementary Fig. 12. Cytotoxicity of STX, STX-binding PS aptamer ($PSAP_{STX}$), and $PSAP_{STX}$ conjugates in C1C12 and PC12 cells. The STX concentration was 45 μM and the $PSAP_{STX}$ concentration was 90 μM. The molar ratio of $PSAP_{STX}$:STX = 2:1. Data are means ± s.d.; n= 4 biological replicates per group. Source data are provided as a Source Data file.
