# Supplementary material for: An aptamer-based depot system for sustained release of small molecule therapeutics
Source: Nat Commun. 2023 Apr 28;14:2444. doi: 10.1038/s41467-023-37002-0 (PMC10147605; doi:10.1038/s41467-023-37002-0)
Supplement: Supplementary file 2 — Reporting Summary [file 41467_2023_37002_MOESM2_ESM.pdf]

nature portfolio

Corresponding author(s): Daniel S. Kohane, Christopher B. Weldon

Last updated by author(s): Feb 17, 2023

Reporting Summary

Nature Portfolio wishes to improve the reproducibility of the work that we publish. This form provides structure for consistency and transparency in reporting. For further information on Nature Portfolio policies, see our Editorial Policies and the Editorial Policy Checklist.

Statistics

For all statistical analyses, confirm that the following items are present in the figure legend, table legend, main text, or Methods section.

n/a	Confirmed	
☐	☒	The exact sample size (*n*) for each experimental group/condition, given as a discrete number and unit of measurement
☐	☒	A statement on whether measurements were taken from distinct samples or whether the same sample was measured repeatedly
☐	☒	The statistical test(s) used AND whether they are one- or two-sided<br>*Only common tests should be described solely by name; describe more complex techniques in the Methods section.*
☒	☐	A description of all covariates tested
☒	☐	A description of any assumptions or corrections, such as tests of normality and adjustment for multiple comparisons
☐	☒	A full description of the statistical parameters including central tendency (e.g. means) or other basic estimates (e.g. regression coefficient) AND variation (e.g. standard deviation) or associated estimates of uncertainty (e.g. confidence intervals)
☐	☒	For null hypothesis testing, the test statistic (e.g. *F*, *t*, *r*) with confidence intervals, effect sizes, degrees of freedom and *P* value noted<br>*Give P values as exact values whenever suitable.*
☒	☐	For Bayesian analysis, information on the choice of priors and Markov chain Monte Carlo settings
☒	☐	For hierarchical and complex designs, identification of the appropriate level for tests and full reporting of outcomes
☒	☐	Estimates of effect sizes (e.g. Cohen's *d*, Pearson's *r*), indicating how they were calculated

Our web collection on statistics for biologists contains articles on many of the points above.

Software and code

Policy information about availability of computer code

Data collection	No software was used.
Data analysis	GraphPad Prism 9 was used for basic statistical analysis and graph production. Origin 2022b was used for plotting. ImageJ (Version 1.53t) was used for image processing. MO. Affinity Analysis (version 2.3) software was used for binding affinity analysis.

For manuscripts utilizing custom algorithms or software that are central to the research but not yet described in published literature, software must be made available to editors and reviewers. We strongly encourage code deposition in a community repository (e.g. GitHub). See the Nature Portfolio guidelines for submitting code & software for further information.

Data

Policy information about availability of data

All manuscripts must include a data availability statement. This statement should provide the following information, where applicable:

- Accession codes, unique identifiers, or web links for publicly available datasets
- A description of any restrictions on data availability
- For clinical datasets or third party data, please ensure that the statement adheres to our policy

The data that support the findings of this study are available within the article and its Supplementary Information files. Source data are provided as a Source Data file. Data are also available from the corresponding author upon request. Source data are provided with this paper.

Human research participants

Policy information about studies involving human research participants and Sex and Gender in Research.

Reporting on sex and gender	N/A
Population characteristics	N/A
Recruitment	N/A
Ethics oversight	N/A

Note that full information on the approval of the study protocol must also be provided in the manuscript.

Field-specific reporting

Please select the one below that is the best fit for your research. If you are not sure, read the appropriate sections before making your selection.

☒ Life sciences ☐ Behavioural & social sciences ☐ Ecological, evolutionary & environmental sciences

For a reference copy of the document with all sections, see nature.com/documents/nr-reporting-summary-flat.pdf

Life sciences study design

All studies must disclose on these points even when the disclosure is negative.

Sample size	We calculated sample size in our studies by using a power analysis. Previous animal studies, or small pilot studies when necessary, served as the basis for calculations of expected averages and deviations used to calculate power, for which we set experiment studies to a value of 0.8. This typically resulted in an experimental group size to be n=4-6, depending on the experiment. Sample size is explicitly stated for each experimental group for individual experiments in figure captions and data descriptions.
Data exclusions	No data were excluded.
Replication	All experimental findings were reliably reproduced. All experiments were performed as technical or biological replications as appropriate for the experiment design. Details of experimental replicates are given in the figure legends. All quantitative measurements (i.e., TTX binding affinity analysis, TTX release, quantification of the fluorescence intensity) have at least three independent repeats.
Randomization	All samples were randomly allocated into experimental groups.
Blinding	All scoring and other histological assessments were performed by an observer blinded as to the nature of the individual samples. No blinding was used for other experiments. The investigators should keep careful track of protocols because that most of the experiments needed multiple treatments (including formulation, cells or rat treatment, sample collection, and so on). Hence, it would be difficult to blind the investigators to group allocation during data collection and analysis.

Reporting for specific materials, systems and methods

We require information from authors about some types of materials, experimental systems and methods used in many studies. Here, indicate whether each material, system or method listed is relevant to your study. If you are not sure if a list item applies to your research, read the appropriate section before selecting a response.

Materials & experimental systems

n/a	Involved in the study	
☒	☐	Antibodies
☐	☒	Eukaryotic cell lines
☒	☐	Palaeontology and archaeology
☐	☒	Animals and other organisms
☒	☐	Clinical data
☒	☐	Dual use research of concern

Methods

n/a	Involved in the study	
☒	☐	ChIP-seq
☒	☐	Flow cytometry
☒	☐	MRI-based neuroimaging

Eukaryotic cell lines

Policy information about cell lines and Sex and Gender in Research

Cell line source(s)	C2C12 mouse myoblasts (American Type Culture Collection (ATCC) CRL-1772) and PC12 rat adrenal gland pheochromocytoma cells (ATCC, CRL-1772).
Authentication	These cell lines were morphologically confirmed.
Mycoplasma contamination	Cells were not tested in our laboratory for mycoplasma contamination.
Commonly misidentified lines (See ICLAC register)	No commonly misidentified cell lines were used.

Animals and other research organisms

Policy information about studies involving animals; ARRIVE guidelines recommended for reporting animal research, and Sex and Gender in Research

Laboratory animals	Adult male Sprague–Dawley rats (Charles River Laboratories), 12-14-week-old, weighing 400-500 g were used.
Wild animals	The study did not involve wild animals.
Reporting on sex	We have shown that there is no difference in the duration of sustained release local anesthetics between male and female rats (Pharm. Res., 2019, 179).
Field-collected samples	The study did not involve samples collected from the field.
Ethics oversight	All procedures associated with animal studies followed recommendations in the Guide for the Care and Use of Laboratory Animals of the National Institute of Health. The Institutional Animal Care and Use Committee (IACUC) at Boston Children's Hospital approved the protocol (protocol 20-07-4222R).

Note that full information on the approval of the study protocol must also be provided in the manuscript.
